# Supplementary material for: Whole-Genome Sequencing Analyses Reveal the Evolution Mechanisms of Typical Biological Features of Decapterus maruadsi
Source: Animals (Basel). 2024 Apr 17;14(8):1202. doi: 10.3390/ani14081202 (PMC11047736; doi:10.3390/ani14081202)
Supplement: Supplementary file 1 [file animals-14-01202-s001.zip › Supplementary Material S1.pdf]

animals

MDPI

Supplementary Materials S1

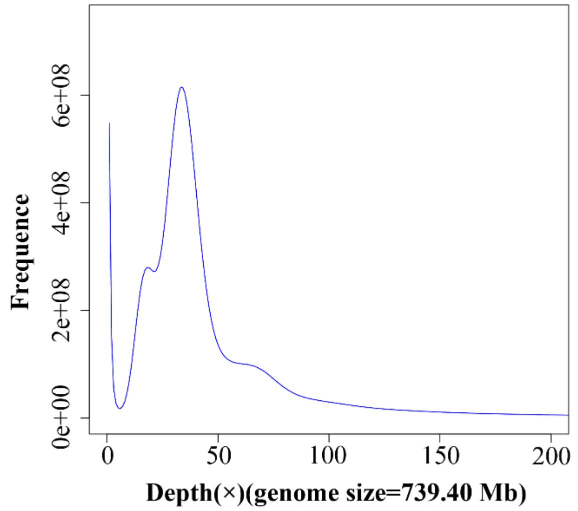

Figure S1. Distribution profiles of 17-mer analysis of Illumina reads.

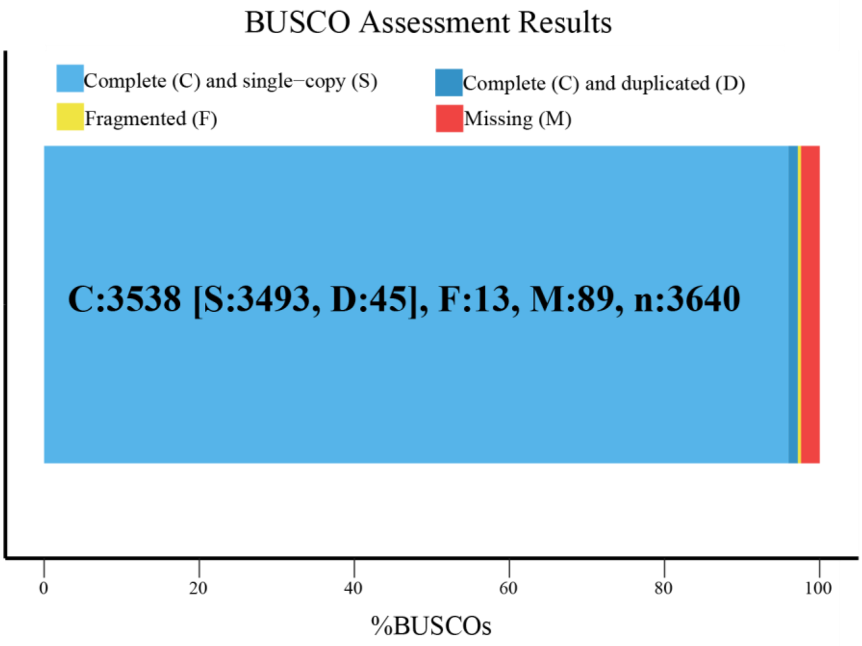

Figure S2. BUSCO assessment results of *D. maruadsi* genome.

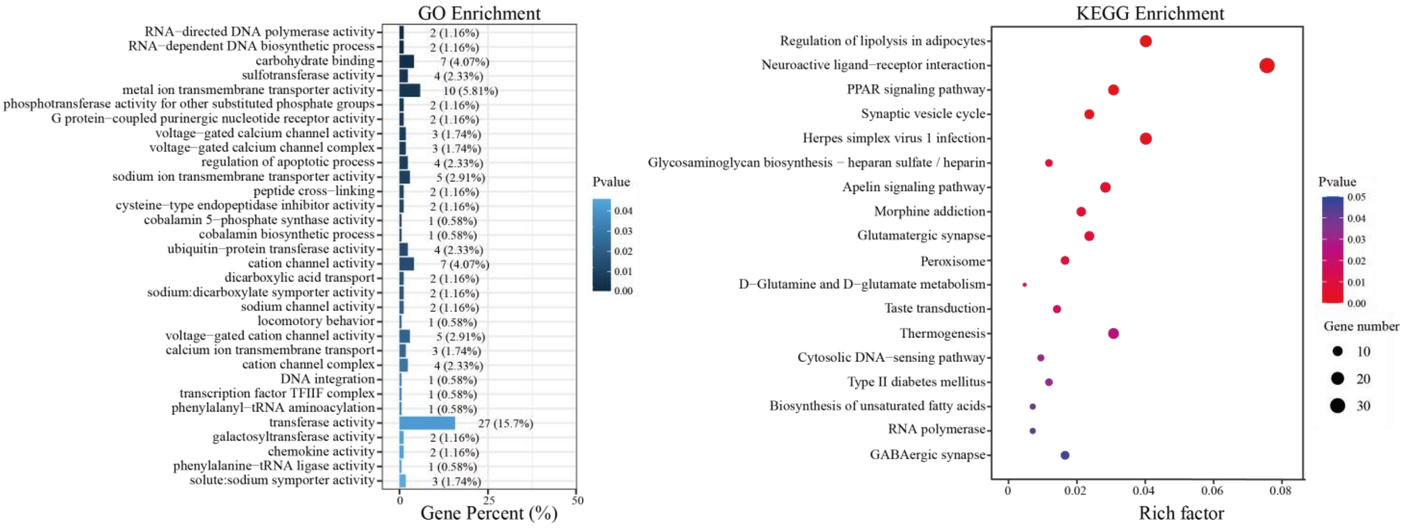

Figure S3. GO and KEGG enrichments of unique gene families to *D. maruadsi*.

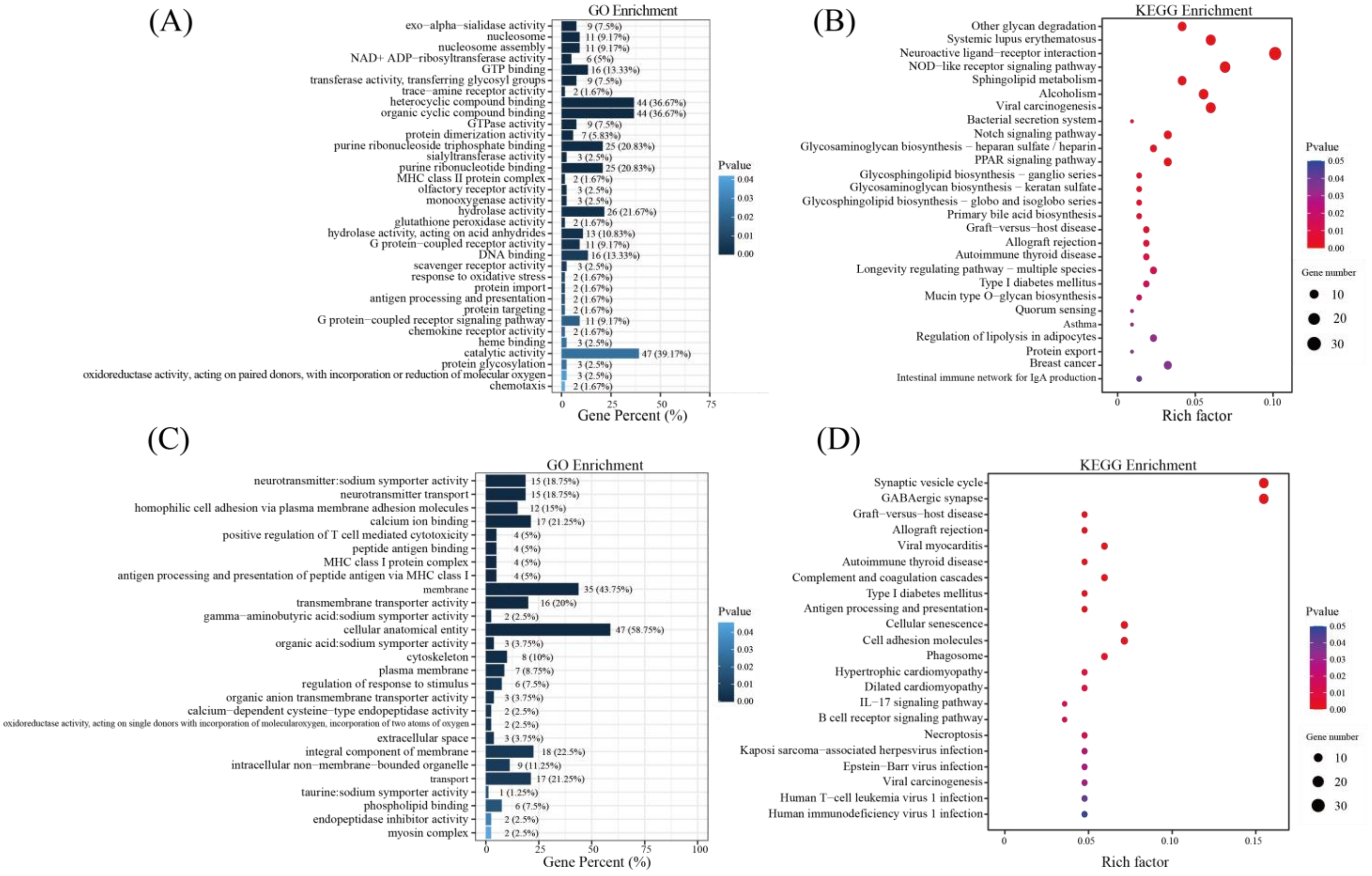

Figure S4. GO and KEGG enrichments of 73 expanded (A and B) and 52 contracted (C and D) gene families in *D. maruadsi*.

Table S1. Types and counts of Di-Tags identified in preliminary Hi-C sequencing data.

Di-Tag Type	Di-Tags Number
Total Di-Tags	6,544,378
Valid Di-Tags	5,564,284
Same circularized Di-Tags	9,091
Same fragment dangling ends Di-Tags	14,054
Same fragment internal Di-Tags	50,650
Re-ligation Di-Tags	173,112
Contiguous Di-Tags	144,769
Wrong size Di-Tags	588,418

Table S2. HiCUP de-duplication, trans, and cis quantity statistics.

Di-Tag Type	All Di-Tags	Unique Di-Tags
Valid Di-Tags	5,564,284	5,176,451
Cis-close Di-Tags (< 10Kbp)	526,555	489,982
Cis-far Di-Tags (> 10Kbp)	2,046,773	1,904,000
Trans Di-Tags	2,990,956	2,782,469

Table S3. Statistics of Illumina RNA-Seq data.

Sample	Raw Reads	Raw Base (G)	Clean Reads	Clean Base (G)	Effective Rate (%)	Error Rate (%)	Q20 (%)	Q30 (%)	GC Content (%)
Heart	21,793,184	6.54	20,817,200	6.25	95.52	0.03	97.69	93.54	49.8
Liver	19,697,155	5.91	18,887,824	5.67	95.89	0.03	97.72	93.55	50.4
Muscle	21,446,521	6.43	20,827,273	6.25	97.11	0.03	97.83	93.85	52.83

Table S4. Repeat sequences in the *D. maruadsi* genome.

RepeatType		length (bp)	Percent
Tandem repeat		72,744,333	10.16%
Interspersed repeat		202,151,366	28.23%
	DNA TE	82,090,490	11.46%
	LINE TE	37,075,462	5.18%
	SINE TE	1,928,796	0.27%
	LTR TE	62,252,932	8.69%
	Other	55	0.000008%
	Unknown	5,046,848	0.70%
Total		274,895,699	38.39%

Table S5. Classifiction and annotation statistics of ncRNA in the *D. maruadsi* genome.

ncRNA type		Copy	Average length (bp)	Total length (bp)	Genome coverage (%)
miRNA		1,829	128.01	234,126	0.032690
tRNA		2,842	75.54	214,675	0.029980
rRNA	rRNA	5,310	112.79	598,939	0.083630
	18S	1,809	102.40	185,249	0.025870
	28S	82	181.27	14,864	0.002076
	5.8S	2	156	312	0.000044
	5S	3,417	116.63	398,514	0.055650
snRNA	snRNA	438	130.53	57,170	0.007983
	CD-box	132	114.02	15,050	0.002101
	HACA-box	104	160.45	16,687	0.002330
	Splicing	188	120.93	22,734	0.003174

Table S6. KEGG enrichment analysis of unique, expanded, and contracted gene families.

**1. Unique (459 gene families, 22 KEGG pathways, P<0.05)**		
**KEGG pathways**	**P-value**	**Genes**
Regulation of lipolysis in adipocytes	2.25E-09	*SAMD3, TSHB, INS, ADCY1, ADORA1*
Neuroactive ligand-receptor interaction	3.93E-09	*GRM4, TSHB, P2YR13, GHRHR, CCR2, CER2, PRSS3P2, F9, GRIN2B*
PPAR signaling pathway	1.16E-06	*SAMD3, UB-EP52*
Synaptic vesicle cycle	0.001071912	*TRNP1, SLC1A1, CACNA1A, SNAP25A, SLC6A13, SLC18A2*
Herpes simplex virus 1 infection	0.001799729	*BCL2L1, MAP6, AUTS2, PCED1A, MR1, ZNF23, ZNP37, ZNF836, MSANTD1*
Glycosaminoglycan biosynthesis - heparan sulfate / heparin	0.003559977	*HS3ST2, HS3ST3B1, KMT5A-B*
Apelin signaling pathway	0.004283059	*SAMD3, ADCY1*
Morphine addiction	0.005512089	*CACNA1A, GABBR2, KCNJ3, PDE1C, ADCY1, ADORA1*
Glutamatergic synapse	0.006400018	*GRM4, SLC1A1, CACNA1A, GRIN2B, KCNJ3, ADCY1, GLS2, GRM7, GABBR2, HTR2A, ADORA1, GRID1*
Peroxisome	8.87E-03	*ADF1, SPCC569.03*
D-Glutamine and D-glutamate metabolism	0.009746174	*GLUD1, GLS2*
Taste transduction	0.015766369	*GRM4, CACNA1A, ASIC2, GABBR2, PED1C*
Thermogenesis	0.024270538	*SAMD3, NPR1, ADCY1*
Cytosolic DNA-sensing pathway	0.029025412	*CARD18, POLR3GL, CCL13*
Type II diabetes mellitus	0.032036904	*INS, CACNA1A*
Biosynthesis of unsaturated fatty acids	0.039633352	*SPCC569.03, TECR*
RNA polymerase	0.042252218	*HI-0712, POLR3GL*
GABAergic synapse	0.046867096	*CACNA1A, GABBR2, ADCY1, GLS2, SLC6A13*
**2. Expansion (73 gene families, 27 KEGG pathways, P<0.05)**		

KEGG pathways	P-value	Genes
Other glycan degradation	1.60E-10	*NEU2, NEU3*
Systemic lupus erythematosus	7.00E-10	*HIST2H2L, H2-Aa*
Neuroactive ligand-receptor interaction	1.91E-07	*LPAR4, TAAR1, TAAR1s, PRSS2, CELA1*
NOD-like receptor signaling pathway	4.82E-07	*GVIN1, GVINP1, PYCARD*
Sphingolipid metabolism	5.89E-07	*NEU2, NEU3*
Alcoholism	6.53E-06	*HIST2H2L, SLC18A2*
Viral carcinogenesis	3.49E-05	*HIST2H2L, CCR3*
Bacterial secretion system	4.55E-04	*SECA*
Notch signaling pathway	4.77E-04	*HES5*
Glycosaminoglycan biosynthesis - heparan sulfate / heparin	4.86E-04	*KMT5AA*
PPAR signaling pathway	1.06E-03	*SAMD3, CYP8B1*
Glycosphingolipid biosynthesis - ganglio series	3.52E-03	*ST3GAL1*
Glycosaminoglycan biosynthesis - keratan sulfate	0.003944136	*ST3GAL1*
Glycosphingolipid biosynthesis - globo and isoglobo series	0.003944136	*ST3GAL1*
Primary bile acid biosynthesis	0.004397271	*CYP8B1*
Graft-versus-host disease	0.004440917	*H2-Aa, PRF1*
Allograft rejection	0.006467993	*H2-Aa, PRF1*
Autoimmune thyroid disease	0.00856437	*H2-Aa, PRF1*
Longevity regulating pathway - multiple species	0.013193295	*HSP30*
Type I diabetes mellitus	0.016651602	*H2-Aa, PRF1*
Mucin type O-glycan biosynthesis	0.017091155	*ST3GAL1*
Quorum sensing	0.027518865	*SECA*
Asthma	0.030028533	*H2-Aa*
Regulation of lipolysis in adipocytes	0.032621052	*SAMD3, NPR1*
Protein export	0.032625497	*SECA*
Breast cancer	0.035453539	*HES5*
Intestinal immune network for IgA production	0.039931832	*BTN1A1, H2-Aa*
**3. Contraction (52 gene families, 22 KEGG pathways, P<0.05)**		
**KEGG pathways**	**P-value**	**Genes**
Synaptic vesicle cycle	5.18E-14	*SLC6A1, SLC6A13, SLC6A11*
GABAergic synapse	1.15E-13	*SLC6A1, SLC6A13, SLC6A11*
Graft-versus-host disease	7.37E-05	*MR1*
Allograft rejection	0.000111569	*MR1*
Viral myocarditis	0.000123025	*MR1, MYH6*
Autoimmune thyroid disease	0.000152551	*MR1*
Complement and coagulation cascades	0.000161749	*A2M, PZP, VWF*
Type I diabetes mellitus	0.000324347	*MR1*
Antigen processing and presentation	0.000533348	*MR1*
Cellular senescence	0.000970876	*MR1, CAPN1*
Cell adhesion molecules	0.001973494	*MR1, VCAM1, CD22*
Phagosome	0.002318501	*MR1, SIGLEC1*
Hypertrophic cardiomyopathy	0.007662597	*TTN*
Dilated cardiomyopathy	0.009824885	*TTN*
IL-17 signaling pathway	0.012604647	*MUC5AC, MUC5B*
B cell receptor signaling pathway	0.01319698	*VCAM1, CD22, SIGLEC1*

Necroptosis	0.013346317	*CAPN1, ALOX5*
Kaposi sarcoma-associated herpesvirus infection	0.02493976	*MR1*
Epstein-Barr virus infection	0.026791833	*MR1*
Viral carcinogenesis	0.027427207	*MR1*
Human T-cell leukemia virus 1 infection	0.042059176	*MR1*
Human immunodeficiency virus 1 infection	0.048056403	*MR1*
